# Innate immunity triggers IL-32 expression by fibroblast-like synoviocytes in rheumatoid arthritis

**DOI:** 10.1186/ar3073

**Published:** 2010-07-08

**Authors:** Ghada Alsaleh, Laetitia Sparsa, Emmanuel Chatelus, Mathieu Ehlinger, Jacques-Eric Gottenberg, Dominique Wachsmann, Jean Sibilia

**Affiliations:** 1 EA3948, Laboratoire Physiopathologie des Arthrites, Université de Strasbourg, UFR Sciences Pharmaceutiques, 74 route du Rhin, 67401 Illkirch, France; 2 Département de Rhumatologie, Hôpitaux Universitaires de Strasbourg, avenue Molière, Strasbourg Hautepierre 67200, France; 3 Département d'Orthopédie, Hôpitaux Universitaires de Strasbourg, Strasbourg Hautepierre 67200, France

## Abstract

**Introduction:**

Interleukin-32 (IL-32) is a recently described cytokine that is a strong inducer of pro-inflammatory cytokines such as tumor necrosis factor (TNF)-α, IL-1β, IL-6, and IL-8. The expression of this cytokine is highly increased in the rheumatoid synovium and correlated with the severity of joint inflammation. Little is known regarding the innate immune-related regulation of IL-32 by fibroblast-like synoviocytes (FLSs). We therefore investigated the effect of innate immune stimulation by ligands of Toll-like receptor (TLR)2, TLR3, and TLR4, and cytokines such as TNF-α and interferon (IFN)-γ, on IL-32 expression by FLSs.

**Methods:**

FLSs were isolated from patients with rheumatoid arthritis (RA) according to the ACR criteria. Quantitative RT-PCR, confocal analysis, and ELISA were performed to evaluate IL-32 mRNA induction and IL-32 release by FLSs stimulated with TLR2 (BLP), TLR3 (poly I:C), and TLR4 (lipopolysaccharide) ligands, TNF-α and IFN-γ.

**Results:**

TLR2, -3, and -4 ligands as well as IFN-γ and TNF-α induced IL-32 β, γ and δ mRNA expression by RA FLSs. Mature IL-32 was expressed intracellularly and released by cells stimulated with the various activators. The IL-32α isoform was expressed intracellularly in response to TNF-α and poly I:C and not released in culture supernatants. Stimulation of FLS with TNF-α, BLP, lipopolysaccharide, or poly I:C concomitant with IFN-γ increased IL-32 expression compared with stimulation with IFN-γ alone.

**Conclusions:**

IL-32 synthesis by FLSs is tightly regulated by innate immunity in rheumatoid arthritis. Thus TNF-α, IFN-γ, double-strand RNA, hyaluronic acid, or other damage-associated molecular patterns (DAMPs), highly secreted in synovial tissues of RA patients, might trigger IL-32 secretion by FLSs. IL-32 might therefore represent a relevant therapeutic target in RA.

## Introduction

Rheumatoid arthritis (RA) is a systemic inflammatory disease that affects predominantly multiple peripheral joints. Although the exact mechanisms that contribute to the pathogenesis are still largely unknown, it is well accepted that numerous inflammatory cells such as T and B cells, fibroblast-like synoviocytes, antigen-presenting cells, and their extensive production of proinflammatory mediators such as TNF-α, IL-1, IL-6, IL-15, IL-17, and IL-18, are implicated [1].

IL-32, a recently described cytokine produced mainly by NK cells, T lymphocytes, epithelial cells, and blood monocytes stimulated by IL-2 or IFN-γ, has recently emerged as an important player in innate immune responses [2,3]. This proinflammatory cytokine is a strong inducer of other proinflammatory cytokines such as TNF-α, IL-1β, IL-6, IL-8, and macrophage inflammatory protein-2 (MIP-2) [3-5]. More recently, it was shown that IL-32 increases IFN-γ production by PBMCs [6,7]. IL-32 might play an important role in inflammatory diseases such as inflammatory bowel diseases and RA [8-10]. IL-32 is highly expressed in RA synovial tissues but not in OA synovial biopsies. Microarray studies in cultured FLSs obtained from patients with RA have shown that the IL-32 gene is one of the most prominently expressed in RA FLSs [11]. Synovial expression of IL-32 is strongly correlated with that of TNF-α and IL-1β but also with the severity of joint inflammation.

Current evidence indicates that FLSs, which constitute the synovial lining, are key actors in pannus formation and the subsequent destruction of cartilage and bone in the joint [12]. Activation of FLSs may be linked either to the cytokine environment, to cell-to-cell contacts, or to interactions between pathogen-associated molecular patterns (PAMPs) or damage-associated molecular patterns (DAMPs) and pattern-recognition receptors (PRRs). Bacterial products, such as lipopolysaccharide (LPS) or peptidoglycan, are known to activate FLSs by interacting with PRRs present on these cells [13,14]. A large number of PRRs, such as TLR2, TLR4, and TLR3, are expressed by FLSs, and their expression is increased in response to inflammatory stimuli [15,16].

FLSs exert a pro-inflammatory activity, essentially by synthesizing cytokines, chemokines, prostanoids, and nitric oxide (NO) [12]. Secretion by FLSs of some cytokines, like IL-6, IL-8, and B-cell-activating factor is regulated by TNF-α, IFN-γ, and PAMPS [17,18]. We therefore investigated the effect of innate immune stimulation by ligands of TLR2, TLR3, TLR4, and cytokines such as TNF-α and IFN-γ, on IL-32 expression by FLSs.

## Materials and methods

### Cell culture

Human FLSs were isolated from synovial tissues from four different RA and OA (osteoarthritis) patients at the time of knee-joint arthroscopic synovectomy, as described previously [19]. The diagnosis conformed to the revised criteria of the American College of Rheumatology [20]. Normal FLSs were isolated from synovial tissues obtained with arthroscopic biopsy. Informed consent was provided according to the Declaration of Helsinki and obtained from all patients. Approval by the ethical committee of the Hopitaux Universitaires de Strasbourg was obtained. FLS cultures were made as previously described [21]. Experiments were performed between the third and the ninth passages. Cell number and cell viability were checked by the MTT test, as described elsewhere [22].

### Stimulation of cells

FLSs (10^6 ^cells) were stimulated with 2 ml of medium alone or medium containing IFN-γ (0.1 ng/ml), TNF-α (10 ng/ml) (R&D Systems, Lille, France), LPS from *Salmonella abortus equi *(Sigma, St. Quentin Fallavier, France) (1 μg/ml), BLP (EMC Microcollections GMBH, Tübigen, Germany) (1 μg/ml), and poly I:C (Invivogen, Toulouse, France) (10 μg/ml). After a 4- or 24-h incubation period, total RNA was extracted by using TRIzol according to the manufacturer's instructions. FLS (2 × 10^5 ^cells) were stimulated with 1 ml of complete medium containing the various activators for 24 h. An IL-32α- specific ELISA test was obtained from Biolegend (Ozyme, Saint Quentin en Yveline, France). IL-32 release was measured with ELISA with the monoclonal antibody KU32-56 as a capture antibody and the biotinylated monoclonal antibody KU32-52 as the detection antibody, according to the manufacturer's instructions (Biolegend, Ozyme, Saint Quentin en Yveline, France). The IL-6-specific ELISA test was from R&D Systems.

### Real-time quantitative RT-PCR

Total RNA isolated from FLSs was reverse transcribed by using the First Strand cDNA Synthesis Kit, according to the manufacturer's instructions (In Vitrogen). Real-time quantitative RT-PCR was performed in a total volume of 20 μl by using a SensiMix Plus SYBR (Quantace; Corbett Life Science, Sydney, Australia) and gene-specific primers:

*IL-32: *5'-TGAGGAGCAGCACCCAGAGC-3'

and 5'-CCGTAGGACTGGAAAGAGGA-3'

*IL-32α: *5'-CTGAAGGCCCGAATGCACCA-3'

and 5'-CCGTAGGACTTGTCACAAAA-3'

*IL-32β: *5'-CTGAAGGCCCGAATGCACCAG-3'

and 5'-GCAAAGGTGGTGTCAGTATC-3'

*IL-32γ: *5'-TGACATGAAGAAGCTGAAGGC-3'

and 5'-CATGACCTTGTCACAAAAGCTC-3'

*IL-32δ: *5'-TCTCTGATGACATGAAGAAGCT-3'

and 5'-GCAAAGGTGGTGTCAGTATC-3'

*GAPDH: *5'-GGTGAAGGTCGGAGTCAACGGA-3'

and 5'-GAGGGATCTCGCTCGCTCCTGGAAGA-3'

*IRF-1*: 5'-AAAAGGAGCCAGATCCCAAGA-3'

and 5'-CATCCGGTACACTCGCACAG-3'

*IRF-3*: 5'-AGCAGAGGACCGGAGCAA-3'

and 5'-AGAGGTGTCTGGCTGGGAAA-3'

IL-32 isoforms were reverse transcribed and amplified. Amplification products were detected as an increased fluorescent signal of SYBRGreen during the amplification cycles. Results were obtained by using SDS Software (Perkin Elmer) and evaluated by using Excel (Microsoft). Melting-curve analysis was performed to assess the specificity of PCR products. Results were normalized to GAPDH and expressed as the fold change compared with samples from cells incubated in medium.

### Immunostaining and confocal microscopy

FLSs (5 × 10^4 ^cells/well; IbiTreat slides) were stimulated with medium containing the various activators. After a 16-h incubation-period, cells were fixed with paraformaldehyde, 4%, at 4°C, washed with PBS, and permeabilized with 0.2% Triton X100 in PBS, pH 7.4, for 10 min. FLSs were incubated with goat anti-IL-32 antibodies (Santa Cruz Biotechnology, Santa Cruz, CA, USA) overnight at 4°C and then with FITC rabbit anti-goat antibodies for 1 h at 25°C. Fluorescence was analyzed with confocal microscopy.

### Detection of cellular IL-32α

FLSs (2 × 10^4 ^cells) were seeded into 96-well plates and then incubated for 16 h and 24 h in 200 μl of complete medium containing the different activators. Cells were then fixed with 4% paraformaldehyde in PBS, pH 7.4, for 20 min. Free aldehyde groups were quenched with NH_4_Cl, 50 m*M*, in PBS, pH 7.4, for 20 min. Nonspecific binding was blocked by incubation in PBS containing 0.2% bovine serum albumin and 0.05% saponin for 30 min at 37°C. The cells were then incubated with biotinylated anti-IL-32α antibodies (Biolegend; Ozyme, Saint Quentin en Yveline, France) for 2 h. Absorbance was measured at 450 nm.

### Transfections

The siRNA duplexes used in our study were designed to target sequences for human *IRF-1 *[GenBank: NM_002198] gene. Four selected siRNA oligonucleotides consisting of sequences of 21 nucleotides were supplied by Dharmacon (Perbio Science, France). Transient transfection of FLSs with siRNA (100 n*M*) was performed by using the Human Dermal Fibroblast Nucleofactor kit from Amaxa, as previously described [23]. FLSs were then plated in 24-well plates (2 × 10^5 ^cells per well). All assays were performed 48 h after transfection. The control was carried out with the Dharmacon siControl nontargeting siRNA consisting of a four-oligonucleotide pool. Transfection efficiency was evaluated with the PmaxGFP control vector.

### Statistical analysis

Values are expressed as mean ± SEM. The significance of the results was analyzed with Wilcoxon's test. The *P *values < 0.05 were considered significant.

## Results

### Effect of IFN-γ and TNF-α on IL-32 synthesis and release by activated RA FLSs

**Figure 1:**
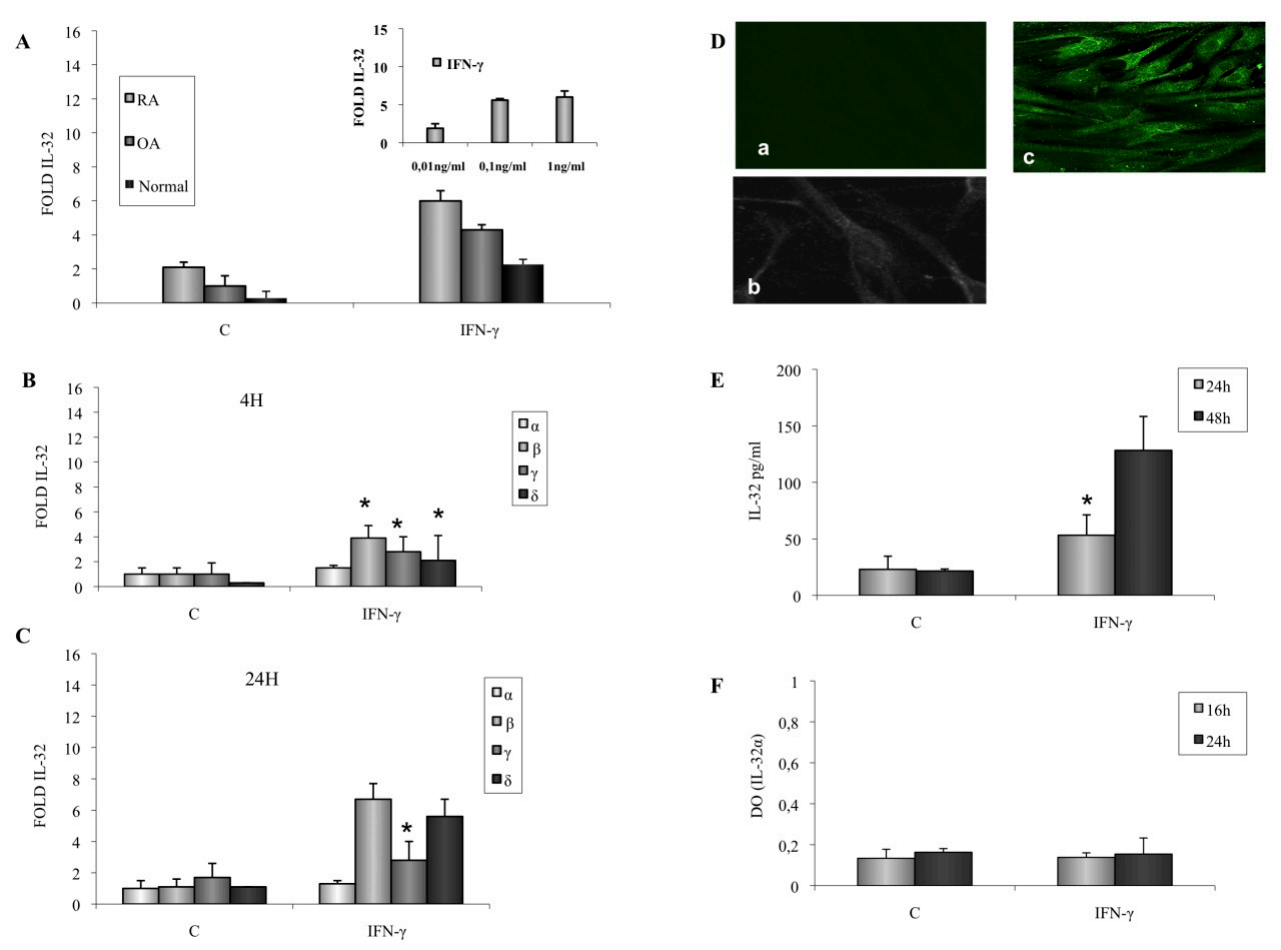
**Effect of IFN-γ (0.1 ng/ml) on IL-32 mRNA expression and IL-32 release by rheumatoid arthritis (RA) fibroblast-like synoviocytes (FLSs)**. **(a) **Dose-dependent effect of IFN-γ (0.01, 0.1, and 1 ng/ml) on IL-32 mRNA expression by RA FLSs activated for 4 h. IL-32 mRNA expression in RA, OA, and normal FLSs activated for 4 h with IFN-γ (0.1 ng/ml). **(b) **IL-32 α, β, γ, and δ mRNA levels were determined by using quantitative RT-PCR in FLSs activated with IFN-γ for 4 h; and **(c) **24 h. **(d) **Confocal analysis of IL-32 expression in FLSs activated for 16 h with IFN-γ **(c) **or medium (a, b). **(e)** IL-32 release in culture supernatants was determined with ELISA after stimulation with IFN-γ or medium (c) for 24 and 48 h. **(f) **Intracellular IL-32α expression was determined with a cell-based ELISA in FLSs activated for 16 and 24 h with IFN-γ or medium **(c)**. Data are expressed as the mean ± SD of three experiments (triplicates) on samples from four different patients with RA. *P < 0.01.

Stimulation of RA FLSs with IFN-γ induced a dose-dependent production of IL-32 transcripts, which were detectable within 4 h (Figure 1a). IL-32mRNA expression was also compared in normal, OA, and RA FLSs activated with 0.1 ng/ml of IFN-γ. As shown in Figure 1a, amounts of IL-32 transcripts were higher in stimulated RA FLSs compared with normal and OA FLSs.

We next investigated the expression of the different isotypes in RA FLSs. Treatment with IFN-γ at a concentration of 0.1 ng/ml resulted in an increasing amount of IL-32β, γ, and δ transcripts, which were detectable within 4 h (Figure 1b), with a mean increase of seven-, three-, and fivefold after stimulation for 24 h, respectively (Figure 1c). IL-32α mRNA was not expressed at 4 and 24 h (Figure 1b, c). To determine whether increased IL-32 mRNA synthesis led to IL-32 protein expression, immunostaining was performed with a polyclonal anti-human IL-32 antibody, which detected all isoforms (α, β, γ, δ). By using confocal microscopy, no basal expression of IL-32 was observed in unstimulated FLS (Figure 1d/a). IL-32 was strongly expressed after a 24-h incubation with IFN-γ (Figure 1d/c). By using an ELISA test that detected IL-32, we observed that IFN-γ induced IL-32 release by FLSs at 24 (55 pg/ml ± 18 pg/ml) and 48 h (125 pg/ml ± 30 pg/ml), compared with nonactivated cells (Figure 1e). The α isoform of IL-32 (IL-32α) protein was neither detected intracellularly in IFN-γ-activated FLSs (Figure 1f) nor released (data not shown).

**Figure 2:**
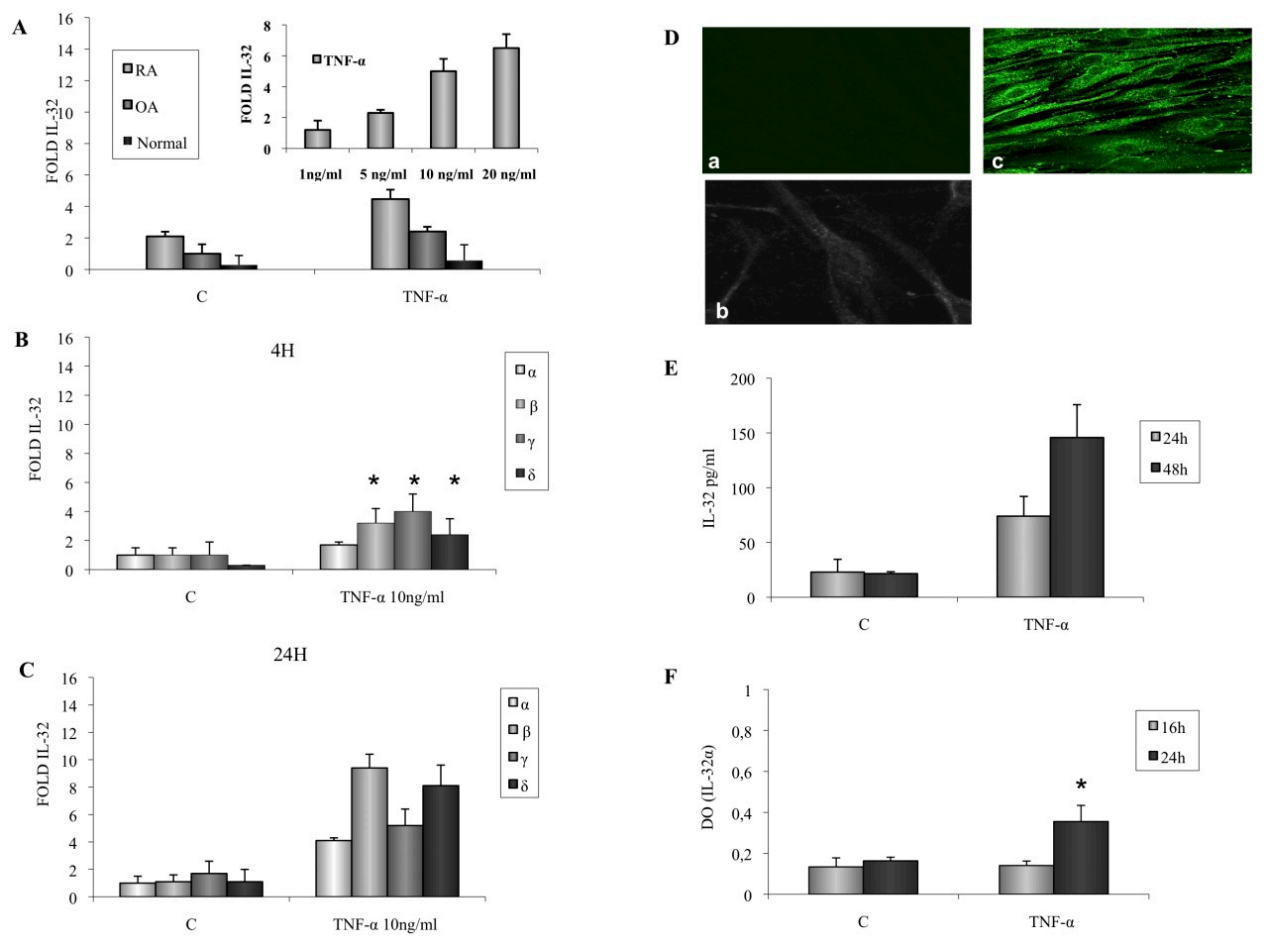
**Effect of TNF-α (10 ng/ml) on IL-32 mRNA expression and IL-32 release by RA fibroblast-like synoviocytes (FLSs)**. **(a) **Dose-dependent effect of TNF-α (1, 5, 10, and 20 ng/ml) on IL-32 mRNA expression by RA FLSs activated for 4 h. IL-32 mRNA expression in RA, OA, and normal FLSs activated for 4 h with TNF-α (10 ng/ml). **(b) **IL-32 α, β, γ, and δ mRNA levels were determined by using quantitative RT-PCR in FLSs activated with TNF-α for 4 h and **(c) **24 h.** (d) **Confocal analysis of IL-32 expression in FLSs activated for 16 h with TNF-α (c) or medium (a, b). **(e)** IL-32 release was determined after stimulation for 24 and 48 h with TNF-α or medium (c) with ELISA.**(f) **Intracellular IL-32α expression was determined with a cell-based ELISA in FLSs activated for 16 and 24 h with TNF-α or medium (c). Data are expressed as the mean ± SD of three experiments (triplicates) on samples from four different patients with RA. *P < 0.01.

Stimulation of RA FLSs with TNF-α induced a dose-dependent production of IL-32 mRNA, which was detectable within 4 h (Figure 2a). By using normal, OA, and RA FLSs activated with 5 ng/ml of TNF-a, we observed that amounts of IL-32 transcripts were higher in stimulated RA FLSs compared with normal and OA FLSs (Figure 2a).

We then assessed the expression of the different isotypes. After stimulation with TNF-α, IL-32α, β, γ, and δ mRNA were detectable within 4 h, with a mean increase of four-, nine-, five- and eightfold after stimulation for 24 h, respectively (Figure 2b, c). Protein expression and release by TNF-α-activated FLSs was demonstrated by immunostaining (Figure 2d/c) and by ELISA (Figure 2e). Mature IL-32α was detected intracellularly (Figure 2f) but not released (data not shown).

### Effect of LPS, BLP, and poly I:C on IL-32 synthesis and release by RA FLSs

**Figure 3:**
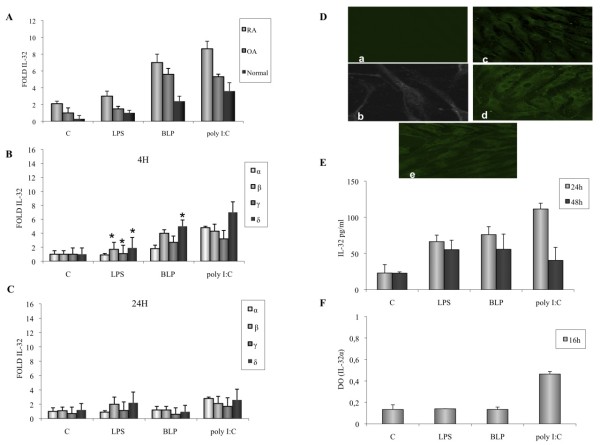
**Effect of TLR2, TLR3, and TLR4 ligands on IL-32 mRNA expression and IL-32 release by RA fibroblast-like synoviocytes (FLSs)**. **(a) **IL-32 mRNA expression in RA, OA, and normal FLSs activated for 4 h with either 1 μg/ml of lipopolysaccharide LPS, or 1 μg/ml of BLP, or 10 μg/ml of poly I:C. **(b) **IL-32 α, β, γ, and δ mRNA levels were determined by using quantitative RT-PCR in FLSs activated with either 1 μg/ml of LPS, or 1 μg/ml of BLP, or 10 μg/ml of poly I:C for 4 h, and **(c) **24 h. **(d) **Confocal analysis of IL-32 expression in FLSs activated for 16 h with either LPS (c), BLP (d), poly I:C (e), or medium (a, b). **(e) **IL-32 release was determined with ELISA after stimulation for 24 and 48 h with either LPS, BLP, poly I:C, or medium (c). **(f) **Intracellular IL-32α expression was determined with a cell-based ELISA in FLSs activated for 16 h with either LPS, BLP, poly I:C, or medium (c). Data are expressed as the mean ± SD of three experiments (triplicates) on samples from four different patients with RA. *P < 0.01.

IL-32 mRNA expression was also compared in normal, OA, and RA FLSs activated with TLR ligands (LPS, BLP, and poly I:C). As shown in Figure 3a, amounts of IL-32 transcripts were higher in stimulated FLSs isolated from RA patients compared with OA and normal FLSs.

IL-32 β, γ, and δ mRNA expression was induced in RA FLSs in response to either BLP or poly I:C after 4 h (Figure 3b), with a lower induction after a 24-h stimulation (Figure 3c). LPS induced only IL-32β and δ isoforms (Figure 3b). An increase (fivefold) of IL-32α mRNA was detected only in poly I:C-activated FLS (Figure 3b, c). Immunostaining and ELISA demonstrated the presence and release of mature IL-32 protein in FLSs stimulated by LPS, BLP, and poly I:C (Figure 3d/c,/d,/e and 3e). IL-32α was also expressed intracellularly in response to poly I:C (Figure 3f) but not released (data not shown).

**Figure 4:**
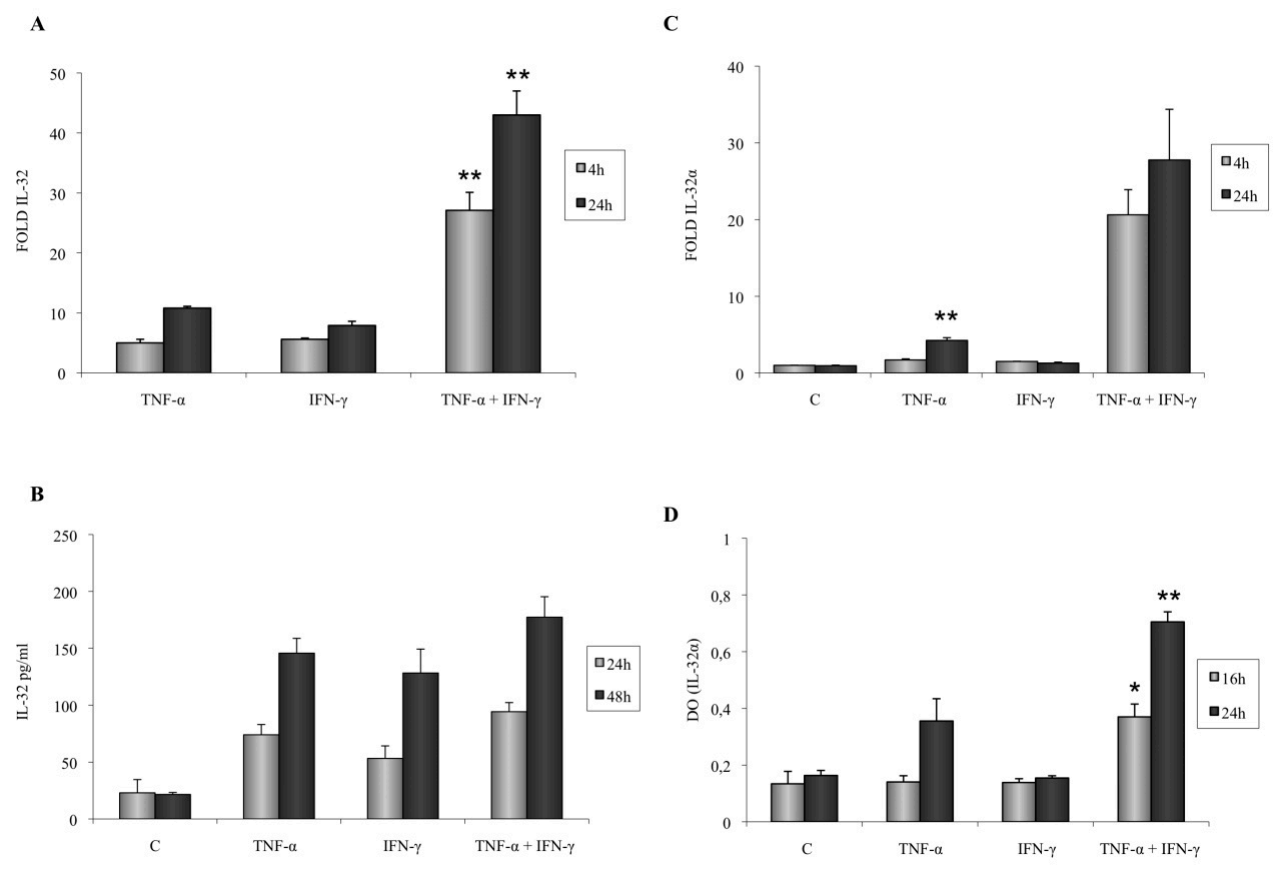
**Effect of co-stimulation of TNF-α (10 ng/ml) and IFN-γ (0.1 ng/ml) on IL-32 mRNA expression and IL-32 release by RA fibroblast-like synoviocytes (FLSs)**. **(a) **FLSs were incubated simultaneously with IFN-γ and TNF-α, and IL-32 mRNA levels were determined after activation for 4 h and 24 h by using quantitative RT-PCR. **(b) **IL-32 release was determined with ELISA in culture supernatants. **(c) **IL-32α mRNA levels were determined by using quantitative RT-PCR in FLSs activated with either IFN-γ and TNF-α or medium (c). **(d) **Intracellular IL-32α expression was determined in IFN-γ + TNF-α- or medium-activated (c) FLSs. Data are expressed as the mean ± SD of three experiments (triplicates) on samples from four different patients with RA. *P < 0.01; **P < 0.001.

The inflammatory environment of the synovial cavity is complex in RA, because most of cytokines are present in the synovial cavity and can interact with each other. We therefore investigated whether a combination of TNF-α (10 ng/ml) and IFN-γ (0.1 ng/ml) could modulate levels of IL-32 mRNA expression by FLSs, because FLS are exposed to both cytokines in the synovium during RA. Concomitant stimulation of FLS with IFN-γ and TNF-α resulted in a strong induction of IL-32 mRNA expression as compared with stimulation with only one of these cytokines. This synergistic effect was observed in IL-32 mRNA 4 h and 24 h after activation with TNF-α and IFN-γ (Figure 4a) but was not observed at the protein level by using ELISA (Figure 4b). However, a concordant effect on mRNA and protein stimulation with both cytokines was demonstrated for the α isoform of IL-32 (Figure 4c, d).

**Figure 5:**
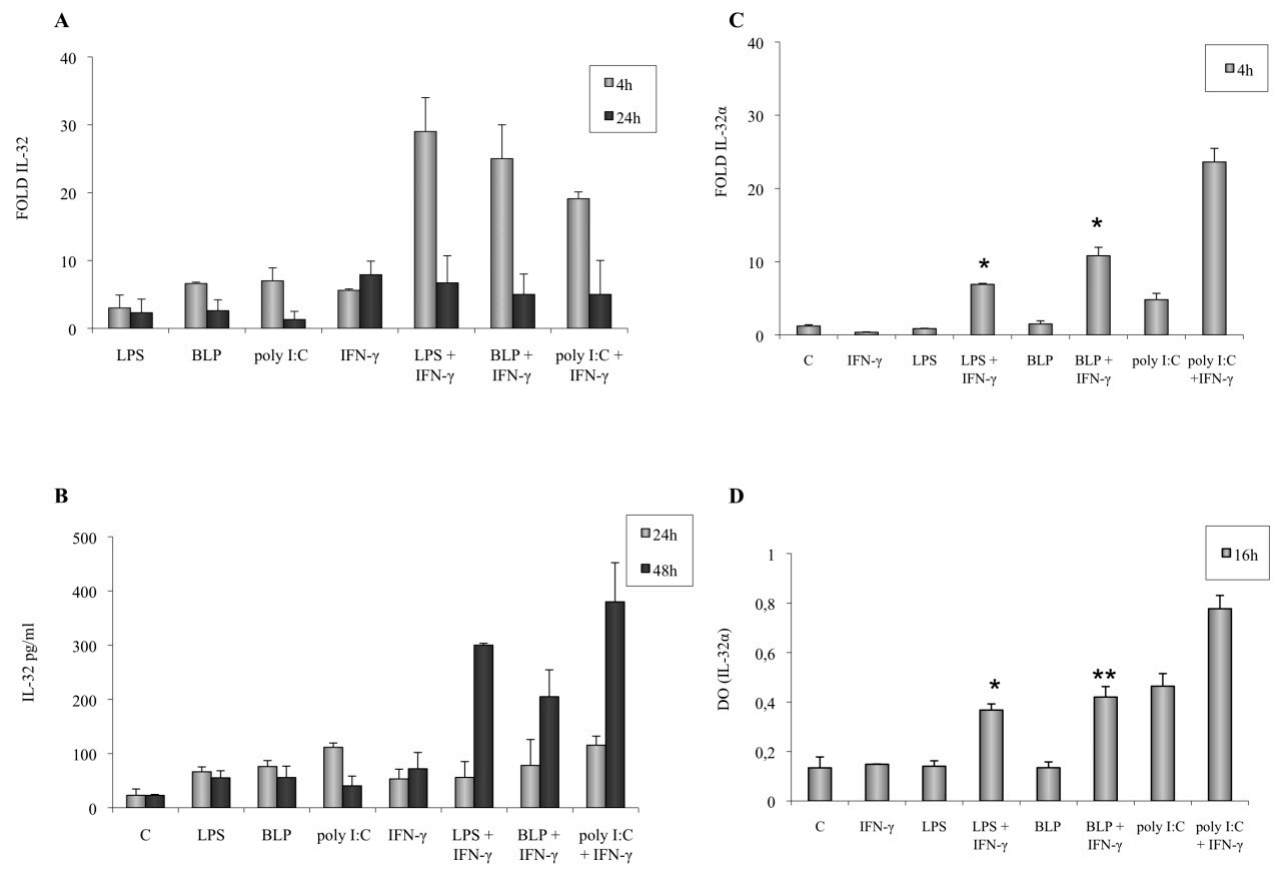
**Effect of co-stimulation of IFN-γ (0.1 ng/ml) with either lipopolysaccharide (LPS; 1 μg/ml), BLP (1 μg/ml), or poly I:C (10 μg/ml) on IL-32 mRNA expression and IL-32 release by RA fibroblast-like synoviocytes (FLSs)**. **(a) **FLSs were incubated simultaneously with IFN-γ and either LPS, BLP, or poly I:C. IL-32 mRNA levels were determined after activation by using quantitative RT-PCR, and IL-32 release was determined with ELISA **(b)**. **(c) **IL-32α mRNA levels were determined with quantitative RT-PCR. **(d) **Intracellular IL-32α expression was evaluated in FLSs with a cell-based ELISA. Data are expressed as the mean ± SD of three experiments (triplicates) on samples from four different patients with RA. *P < 0.01; **P < 0.001.

We also studied the effect of a concomitant stimulation of IFN-γ (0.1 ng/ml) and either BLP, LPS, or poly I:C. Stimulation of FLS with IFN-γ (0.1 ng/ml) and either of these PAMPs strongly induced the transcript levels of IL-32 mRNA as compared with FLSs activated with the same amount of IFN-γ alone (Figure 5a). This effect was observed after 4 h of stimulation but not after 24 h. A synergistic effect on IL-32 release was observed after activation with IFN-γ and either LPS, BLP, or poly I:C (Figure 5b). Moreover, LPS, BLP, and poly I:C also exert a synergistic effect on IL-32α mRNA synthesis and IL-32α intracellular expression, when combined with IFN-γ (Figure 5c, d).

### Role of IRF-1 in the synergistic induction of IL-32

**Figure 6:**
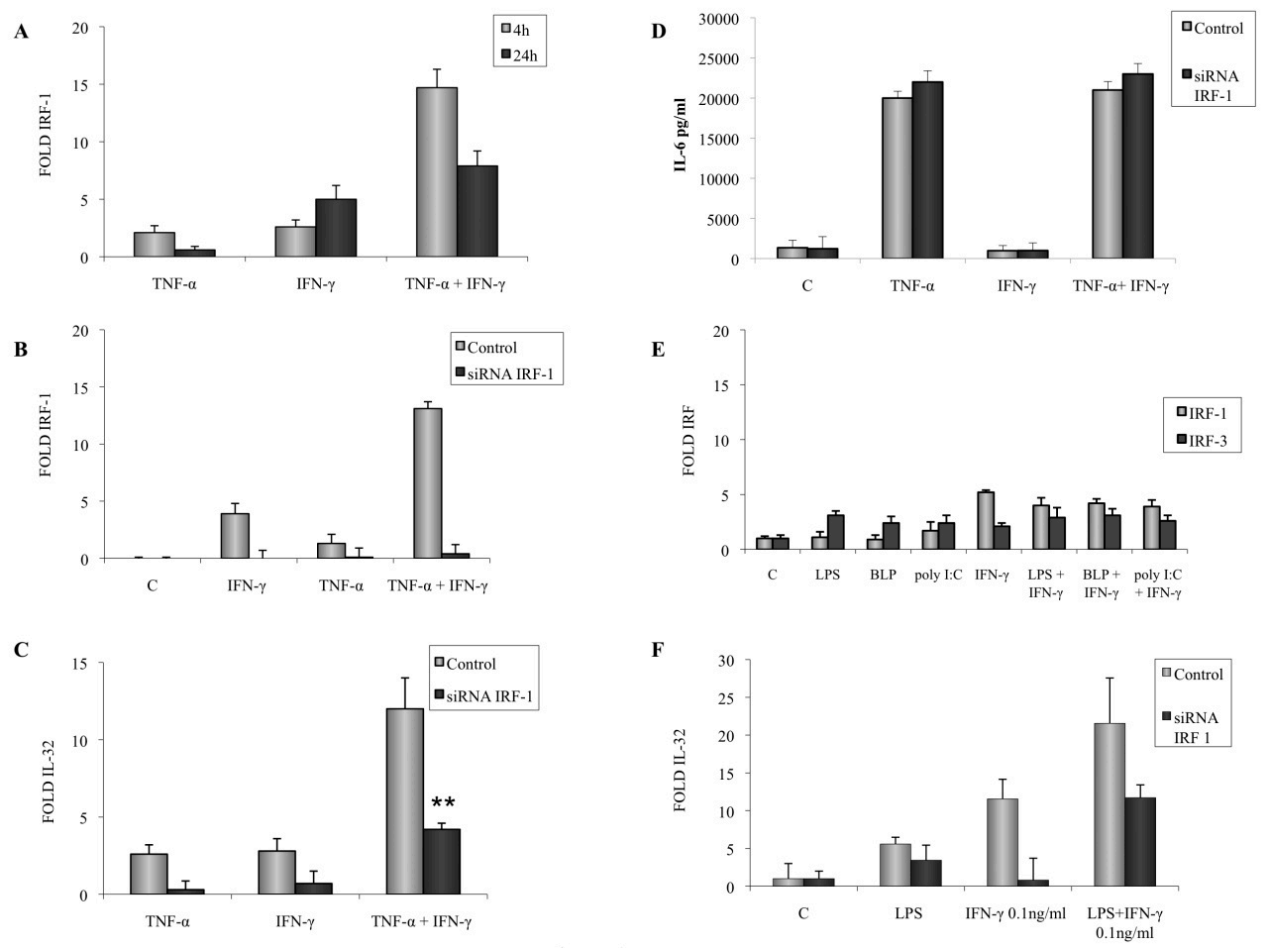
**Effect of co-stimulation of either TLR ligands or TNF-α (10 ng/ml) and IFN-γ (0.1 ng/ml) on IRF-1 and IRF-3 mRNA expression**. **(a) **Fibroblast-like synoviocytes (FLSs) were incubated simultaneously with IFN-γ and TNF-α, and IFN regular factor (IRF-1) mRNA expression was assessed after activation (4 and 24 h) by using quantitative RT-PCR. **(b, c) **Rheumatoid arthritis (RA) FLSs were transfected with IRF-1 antisense molecules or with a negative control (Control). Concomitant activation with IFN-γ and TNF-α of transfected RA FLSs was performed 24 h after transfection, for 4 h. IRF-1 (b) IL-32 (c) expression was determined with quantitative RT-PCR. **(d) **IL-6 release was determined with ELISA after co-stimulation with IFN-γ and TNF-α. **(e) **FLSs were incubated with IFN-γ or with IFN-γ and either LPS (1 μg/ml), BLP (1 μg/ml), or poly I:C (10 μg/ml) for 4 h. IFN regular factor (IRF-1) and IRF-3 mRNA expression was determined by using quantitative RT-PCR. **(f) **RA FLSs were transfected with IRF-1 antisense molecules or with a negative control (Control). Concomitant activation with IFN-γ and LPS of transfected RA FLSs was performed 24 h after transfection, for 4 h. IL-32 expression was determined with quantitative RT-PCR. Data are expressed as the mean ± SD of three experiments (triplicates) on samples from four different patients with RA. **P < 0.001.

We subsequently analyzed the mechanisms responsible for the synergistic effect of PAMPs or TNF-α on IFN-γ related IL-32 induction. Previous studies showed that nuclear concentrations of IFN regulatory factor-1 (IRF-1) were found to increase after stimulation with IFN-γ and TNF-α compared with stimulation with individual cytokines [24,25]. We therefore evaluated IRF-1 mRNA expression in RA FLSs, stimulated with either TNF-α or IFN-γ alone or in combination. Stimulation with IFN-γ induced IRF-1 mRNA expression in activated RA FLSs, and concomitant stimulation of FLSs with IFN-γ and TNF-α resulted in a stronger increase of IRF-1 mRNA expression (Figure 6a). These results indicate that a simultaneous stimulation with TNF-α has a synergistic effect on IFN-γ-induced IRF-1 transcription in FLSs.

To determine whether IRF-1 is necessary for IL-32 mRNA synthesis by stimulated FLSs, cells were transfected with siRNA targeting IRF-1 or control siRNA for 48 h and then stimulated with TNF-α and IFN-γ alone or in combination. Transfection with siRNAs did not affect cell viability, assessed by the MTT test. We first confirmed that transfection of siRNA targeting IRF-1 impaired endogenous IRF-1mRNA expression, as compared with IRF-1 expression in cells transfected with a nontargeting, control siRNA (Figure 6b). Inhibition of IRF-1 significantly reduced IL-32 mRNA expression after TNF-α, IFN-γ, and TNF-α + IFN-γ stimulation of FLSs (Figure 6c). This role of IRF-1 in the synergy between TNF-α and IFN-γ was specific to IL-32, because the release of IL-6, another proinflammatory cytokine, was not modified after inhibition of IRF-1 (Figure 6d). Stimulation with either LPS, BLP or poly I:C did not induce IRF-1 mRNA expression but IRF-3 mRNA (Figure 6e), and no synergic effect either on IRF-1 or IRF-3 mRNA expression was observed when FLSs were concomitantly stimulated with IFN-γ and LPS, BLP, or poly I:C (Figure 6e). Likewise, by using IRF-1 siRNA, the synergy between LPS and IFN-γ was not modified (Figure 6f), converse to that observed after stimulation with IFN-γ and TNF-α. Thus, these results indicate that IRF-1 is involved in the synergistic effect of TNF-α and IFN-γ on IL-32 mRNA expression by activated FLSs, but IRF-1 and IRF-3 are not implicated in the synergy for the induction of IL-32 observed between IFN-γ and the PAMPs studied.

## Discussion

The synovial expression of IL-32, a potent proinflammatory cytokine, is increased in RA and correlated with disease activity [10]. A recent study demonstrated that resident cells of joints, FLSs, which secrete proinflammatory cytokines such as IL-6 and IL-8 but not TNF-α or IL-1β, secrete high levels of IL-32 [26,27]. The expression of cytokines by FLSs is regulated, at least partly, by innate immunity. However, little is known regarding the innate-immune-related regulation of IL-32 by FLSs. We demonstrated that proinflammatory cytokines involved in the pathogenesis of RA, as well as stimulation of various TLR receptors, result in the expression of IL-32 by FLSs, key target and resident cells of RA. Moreover, a synergistic interaction between IFN-γ and PAMPs for IL-32 induction was observed. We demonstrated that synergy between TNF-α and IFN-γ was related to the induction of IRF-1.

We first confirmed studies from Mun *et al. *[26] and Shoda *et al. *[27], demonstrating that activation of FLSs with TNF-α caused IL-32 synthesis and release. The ability of IFN-γ to induce IL-32 production in epithelial cells and monocytes was previously reported by Kim *et al. *[3]. IFN-γ is produced in RA by either by CD4^+ ^T cells, or by subsets of CD8^+ ^CD40L T cells or CD4^+^T cells, which express KIR2DS2 and NKG2D receptors. Immunohistochemical studies have shown the reinforced expression of Stat proteins in rheumatoid synovial tissues, which suggests the importance of the IFN-γ/JAK/Stat pathway [28,29]. Moreover, under most conditions in RA, IFN-γ release correlates with TNF-α production [30]. We therefore assessed the role of IFN-γ in IL-32 synthesis and secretion by FLSs. Exposure to IFN-γ increased IL-32 mRNA transcription and protein release, as demonstrated for TNF-α. In RA FLSs, IFN-γ is unlikely to function as a direct inducer of proinflammatory cytokines synthesis, such as that of TNF-α, IL-6, and IL-8 [17]. Thus, these data suggest that IFN-γ, by means of IL-32 release, might play an important role in the amplification of inflammatory reactions in RA.

An important issue relevant to this study is represented by the induction of IL-32 mRNA transcription in response to LPS, BLP, and poly I:C. This effect was particularly significant in response to BLP and poly I:C at 4 h, with a decrease at 24 h corresponding to a kinetic different from the one obtained with IFN-γ and TNF-α. However, this is frequently observed with certain cytokines such as TNF-α. These findings are not concordant with results from Netea's group [7] on activated PBMCs, showing that TLR2 and TLR3 ligands did not induce an increase in IL-32 release. In keeping with their results, we also observed that LPS was a moderate inducer of IL-32 expression in RA FLSs.

IL-32 is transcribed as six alternative splice variants. Splice variants are quite unusual for cytokines, but they exist in other cytokines such as IL-15 and IL-1F7. The four spliced variants are expressed in TNF-α-stimulated RA FLSs, but their respective roles in RA pathogenesis remain to be determined [31]. In this study, we observed that β, γ, and δ isoforms were transcribed in FLSs activated with either TNF-α, IFN-γ, BLP, or poly I:C. The β and δ isoforms were moderately induced by LPS.

Interestingly, we demonstrated that IL-32α mRNA is upregulated in response to TNF-α and poly I:C and that this variant is only cell associated in FLSs and never released. This is in agreement with a study in PBMCs showing that IL-32α expression is upregulated in response to *Mycobacterium tuberculosis *and remained cell associated [7]. This effect might depend on cell-type, because IL-32α can be released by some epithelial cells lines in response to IFN-γ, TNF-α and IL-1β [9].

IFN-γ and TNF-α are cytokines characterized by complex reciprocal effects. They synergize to increase collagen synthesis by dermal fibroblasts or glycosaminoglycans synthesis by lung fibroblasts, and they are tightly involved in the inflammatory response during septic shock [32,33]. An important result is that IRF-1 is required for the synthesis of IL-32 by TNF-α. Its role in IFN-γ signaling is well known, but not in TNF-α signaling. We also showed that this effect is specific, as IRF-1 does not play any role in IL-6 synthesis. Of note in the present study, TNF-α exerts a synergistic effect on IFN-γ- induced IL-32 mRNA, which is related to IRF-1 upregulation. The expression of IL-32 mRNA was considerably reduced (70%) after inhibition of IRF-1 in FLSs activated with a combination of TNF-α and INF-γ.

Unexpectedly, release of IL-32 protein, was not increased after TNF-α and IFN-γ stimulation, but the intracellular expression of the IL-32α was upregulated. Similar results were obtained in epithelial cells, in which a combination of TNF-α and INF-γ increased IL-32α expression [9]. Knowledge of the role of intracellular IL-32 is still limited, but IL-32α may play a role as a cytoplasmic protein. Recently it was demonstrated that IL-32α acts synergistically with NOD-specific peptidoglycans for the release of inflammatory cytokines [5].

Concomitant stimulation with IFN-γ and other TLR ligands also increased IL-32 mRNA expression and protein release, but the mechanism involved in these synergies remains to be identified. We demonstrated that they are not related to IRF-1, converse to the synergy between IFN-γ and TNF-α. Thus, these concomitant stimulations did not upregulate IRF-1, and IRF-1 silencing did not inhibit the synergy between IFN-γ and PAMPs for IL-32 induction. A hallmark of tissue injury is the turnover of extracellular matrix components, which can subsequently act as DAMPs. Increased accumulation of fragmented hyaluronan was noticed in several autoimmune diseases. Hyaluronan fragments signal through TLR2 and TLR4 in macrophages, and tenascin-C activates TLR4 in macrophages and FLSs [34,35]. Moreover, RNA release from necrotic cells acts as and endogenous TLR3 ligand for the stimulation of proinflammatory cytokines release [36]. Therefore, our data raise the possibility that triggers, including bacterial components, of endogenous ligands may promote IL-32 synthesis and release by activating TLR pathways.

## Conclusions

IL-32 is known to activate the p38MAPK and the NF-κB signal-transduction pathways and to induce the expression of other proinflammatory cytokines, such as TNF-α, IL-1β, and IL-18, partly by amplifying the signals induced by NOD2 [5,7]. Because these cytokines are never released by activated FLSs, the present data suggest that FLSs may play an important role in the amplification of inflammatory reactions in RA, by expressing IL-32, which in turn induces the expression of TNF-α, IL-1, or IL-18 by macrophages or dendritic cells, bridging innate and adaptive immunity. IL-32 might therefore represent a relevant therapeutic target in RA.

## Abbreviations

DAMPs: damage-associated molecular patterns; FLS: fibroblast-like synoviocyte; IRF-1: IFN regular factor; LPS: lipopolysaccharide; PAMPs: pathogen-associated molecular patterns; PBMCs: peripheral blood mononuclear cells; PRRs: pattern-recognition receptors; RA: rheumatoid arthritis.

## Competing interests

The authors declare that they have no competing interests.

## Authors' contributions

GA and LS designed and performed all experiments and drafted the manuscript. EC and ME assisted in designing the study. JS conceived the study. JEG edited the manuscript. DW conceived the study and drafted and edited the manuscript. All authors read and approved the final manuscript.
